# Bioengineered exosomal-membrane-camouflaged abiotic nanocarriers: neurodegenerative diseases, tissue engineering and regenerative medicine

**DOI:** 10.1186/s40779-023-00453-z

**Published:** 2023-04-27

**Authors:** Daniela Lopes, Joana Lopes, Miguel Pereira-Silva, Diana Peixoto, Navid Rabiee, Francisco Veiga, Omid Moradi, Zhan-Hu Guo, Xiang-Dong Wang, João Conde, Pooyan Makvandi, Ana Cláudia Paiva-Santos

**Affiliations:** 1 Department of Pharmaceutical Technology, Faculty of Pharmacy of the University of Coimbra, University of Coimbra, 3000-548 Coimbra, Portugal; 2 REQUIMTE/LAQV, Group of Pharmaceutical Technology, Faculty of Pharmacy of the University of Coimbra, University of Coimbra, 3000-548 Coimbra, Portugal; 3 School of Engineering, Macquarie University, Sydney, NSW 2109 Australia; 4 Centre for Molecular Medicine and Innovative Therapeutics, Murdoch University, Perth, WA 6150 Australia; 5 Department of Chemistry, Shahr-e-Qods Branch, Islamic Azad University, Tehran, 374-37515 Iran; 6 Integrated Composites Laboratory (ICL), Department of Mechanical and Construction Engineering, Northumbria University, Newcastle Upon Tyne, NE1 8ST UK; 7 Department of Pulmonary and Critical Care Medicine, Zhongshan Hospital, Fudan University Shanghai Medical College, Shanghai, 200032 China; 8 Faculdade de Ciências Médicas, NOVA Medical School, Universidade Nova de Lisboa, 1169-056 Lisbon, Portugal; 9 Centre for Toxicogenomics and Human Health, Genetics, Oncology and Human Toxicology, Faculdade de Ciências Médicas, NOVA Medical School, Universidade Nova de Lisboa, 1169-056 Lisbon, Portugal; 10 School of Engineering, Institute for Bioengineering, The University of Edinburgh, Edinburgh, EH9 3JL UK

**Keywords:** Biomimetic, Cell membrane coating, Exosome, Exosomal-membrane-coated nanoparticle, Extracellular vesicle (EV)

## Abstract

A bio-inspired strategy has recently been developed for camouflaging nanocarriers with biomembranes, such as natural cell membranes or subcellular structure-derived membranes. This strategy endows cloaked nanomaterials with improved interfacial properties, superior cell targeting, immune evasion potential, and prolonged duration of systemic circulation. Here, we summarize recent advances in the production and application of exosomal membrane-coated nanomaterials. The structure, properties, and manner in which exosomes communicate with cells are first reviewed. This is followed by a discussion of the types of exosomes and their fabrication methods. We then discuss the applications of biomimetic exosomes and membrane-cloaked nanocarriers in tissue engineering, regenerative medicine, imaging, and the treatment of neurodegenerative diseases. Finally, we appraise the current challenges associated with the clinical translation of biomimetic exosomal membrane-surface-engineered nanovehicles and evaluate the future of this technology.

## Background

Nanomaterials have the potential to be used to diagnose and treat various human diseases due to their unique ability to deliver therapeutic bioactive molecules to target sites [1–4]. Treatment strategies utilizing nanomaterials have demonstrated improved efficacy and safety when compared to conventional therapies [5–9]. Despite the many potential applications of nanoparticles (NPs) in medicine, their clinical use is limited owing to their poor biocompatibility and inability to cross biological barriers. Due to their foreign nature, abiotic nanomaterials are rapidly cleared by the body’s mononuclear phagocyte system, resulting in a short duration of systemic circulation and reduced delivery efficacy to target sites [10].

To circumvent these hurdles, recent studies have focused on camouflaging abiotic NPs with biological cell membranes, such as those of red blood cells [11], white blood cells [12], platelets [13], stem cells [14], or cancer cells [15], to improve in vivo interactions and biofunctionality. This involves functionalizing the surface of NPs with a cell membrane via top-down approaches [12, 16]. This promising cell-mimicking approach enables NPs to acquire the inherent biological properties of progenitor cell membranes. By covering NPs with a natural cell membrane, the antigenic profile and interfacial properties of the progenitor cell can be faithfully preserved and transferred to the abiotic NPs [4, 17].

**Fig. 1:**
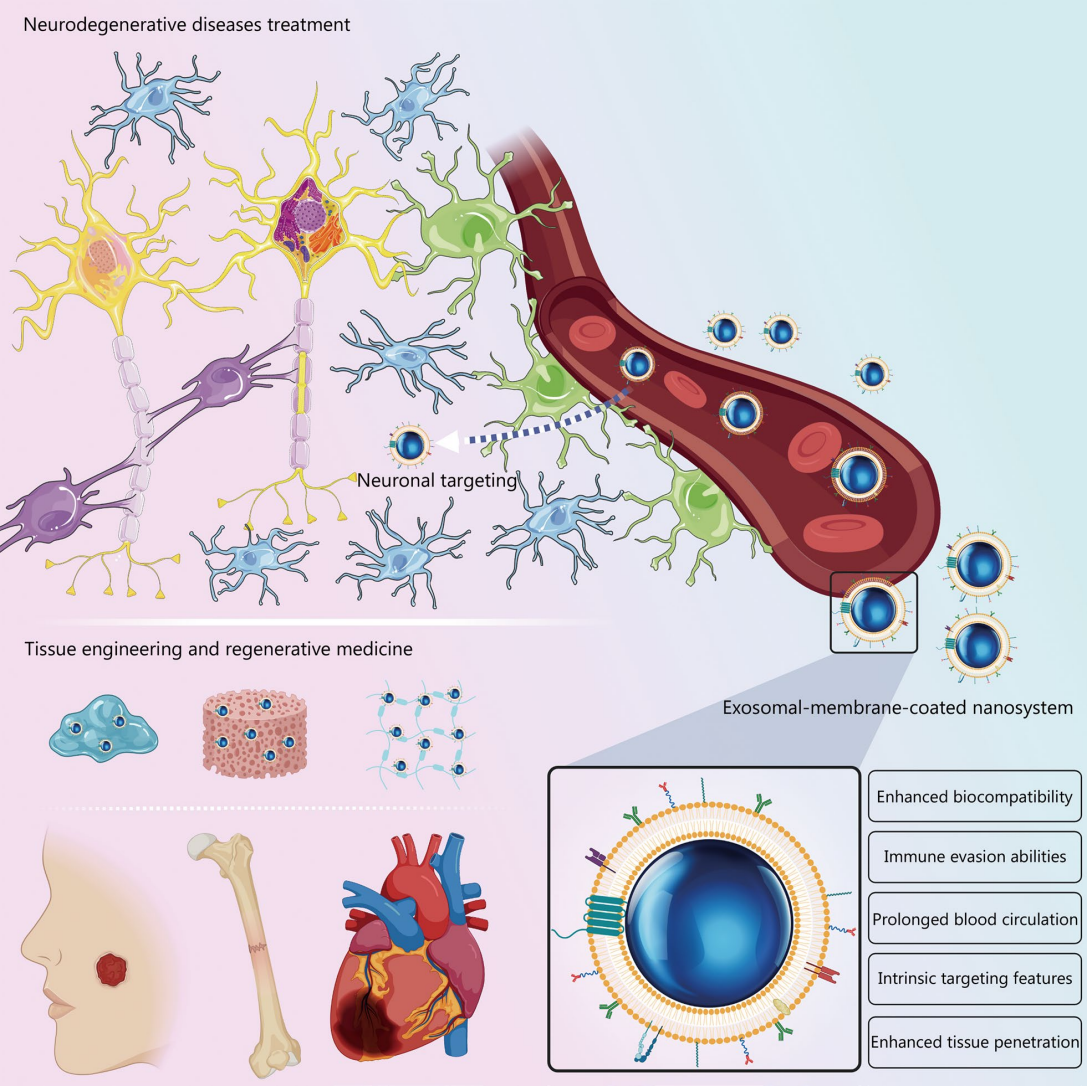
Exosomal-membrane-coated nanosystems are promising nanotechnological tools for biomedical applications. Depiction of the biological benefits of exosomal-membrane-coated nanosystems, including intrinsic tissue-targeting and tissue-specific accumulation features, prolonged blood circulation, and enhanced biocompatibility, stability, and immune evasion abilities, as well as their applications in biomedical settings

Another variety of biomimetic and nature-inspired technologies uses membranes of subcellular structures. Recently, exosomal membranes have attracted considerable interest for use as nanomaterial coatings [18, 19]. Exosomes are produced by cells and have optimal nanoscale sizes. Exosome membranes are more biomimetic than synthetic membranes when used to coat NPs. Membrane extraction from exosomes does not require aggressive techniques such as extrusion or sonication, which are often employed for cell membrane extraction and nanovesicle derivation. Exosomes are excellent intercellular messengers optimized for intercellular communication and interaction [20, 21]. For these reasons, coating NPs with the membranes of naturally secreted exosomes offers many advantages over the use of natural cell membranes. These advantages include intrinsic targeting, cell-specific uptake, prolonged systemic circulation, enhanced biocompatibility, stability, and immune evasion. Exosomal membrane-coated NPs have demonstrated exciting results, improving therapeutic efficacy and reducing off-target toxicity in healthy tissues (Fig. 1) [18, 19, 21].

The present review provides an overview of the most recent research and current advances in exosomal-membrane-coated nanomaterials. We first give an overview of the composition, mechanisms of biogenesis, and biological functions of natural exosomes, then discuss the fabrication of exosomal-membrane-functionalized NPs. Next, we review the biomedical applications of these biomimetic nanostructures, including tissue regeneration and the diagnosis and treatment of neurodegenerative diseases (Fig. 1). Finally, we discuss the major challenges for successfully implementing this technology in clinical settings and our perspectives on the future of this emerging biomimetic coating approach.

## Exosomes: structure, properties, and cell communication

According to the Minimal Information for Studies of Extracellular Vesicles (MISEV) 2018 guidelines proposed by the International Society for Extracellular Vesicles (ISEV), extracellular vesicles (EVs) are defined as natural, cell-secreted vesicles bound by a phospholipid bilayer that are unable to replicate as they do not contain a functional nucleus [22].

Exosomes are a small subtype of cell-secreted EVs of endocytic origin, ranging from 30 to 150 nm in size [23, 24]. They function as mediators of cell–cell communication by delivering a wide range of biological components, such as proteins, lipids, and nucleic acids, to neighboring and distant cells. Exosomes are thus important messengers in intercellular communication [25]. Because of their ability to transport biomolecules between surrounding and distant cells, exosomes can mediate short- and long-distance cell–cell communication and influence various physiological and pathological functions of recipient cells [20].

### Structure and physiology of exosomes

**Fig. 2:**
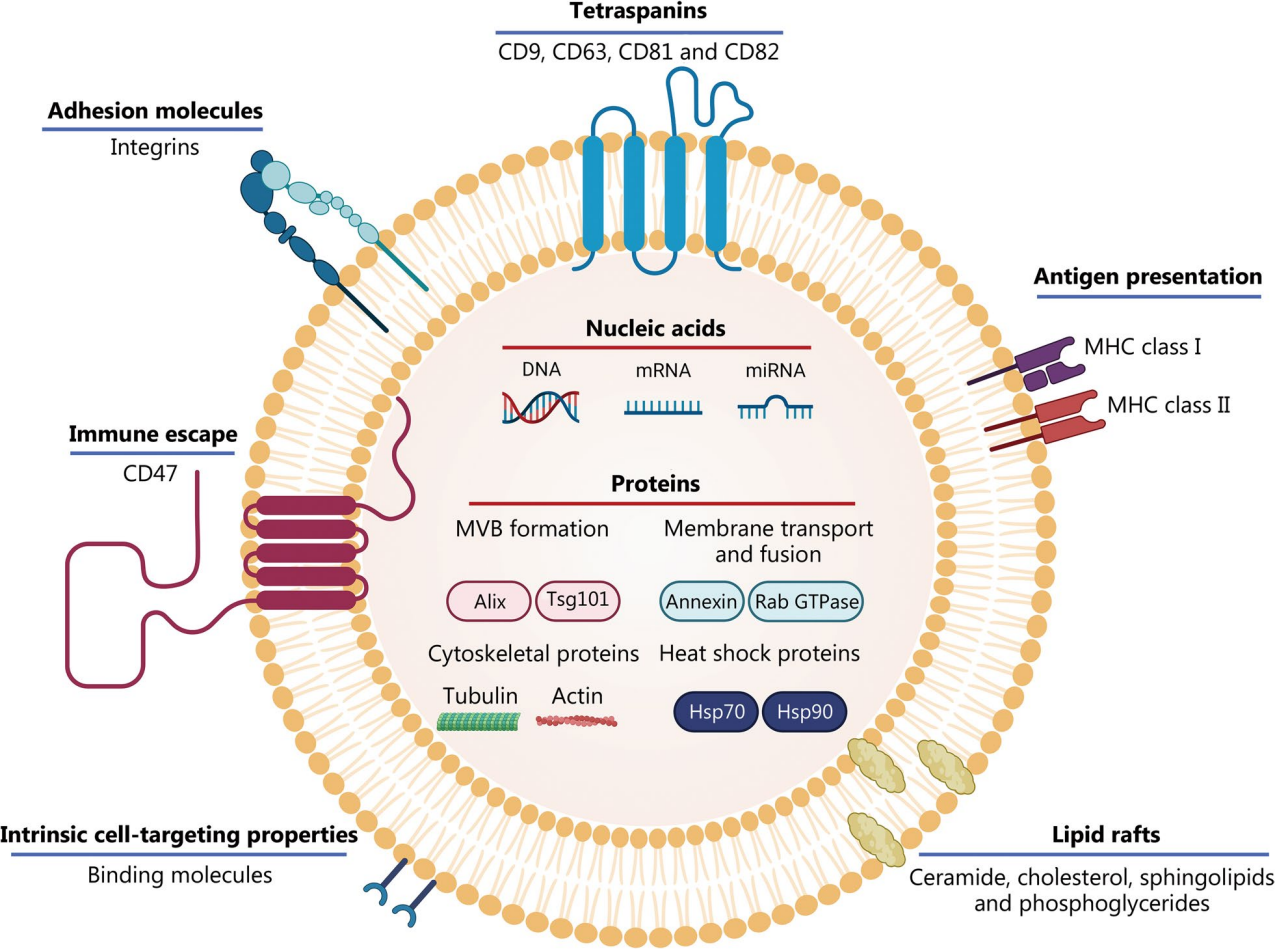
Exosome structure and composition. Exosomes are enriched with lipid rafts, nucleic acids, and proteins. The latter include adhesion molecules, tetraspanins (e.g., CD9, CD63, CD81, and CD82), proteins responsible for membrane transport and fusion (e.g., annexin and Rab GTPase), proteins involved in MVB biogenesis (e.g., Alix and Tsg101), heat shock proteins (e.g., Hsp70 and Hsp90), and cytoskeletal proteins (e.g., actin and tubulin). MHC class I and class II proteins may also be found in exosomes. Alix apoptosis-linked gene-2 interacting protein X, DNA deoxyribonucleic acid, Hsp heat shock protein, MHC major histocompatibility complex, miRNA microRNA, mRNA messenger RNA, MVB multivesicular body, Tsg101 tumor susceptibility gene 101

Similar to synthetic liposomes, exosomes have an amphiphilic structure consisting of an aqueous core surrounded by a phospholipid bilayer [26]. As shown in Fig. 2, exosomes are mainly composed of a diverse set of proteins [27], lipids [28], and nucleic acids [29]. The biological contents of an exosome resemble the composition of the cell that secreted it. As a result, exosome composition is directly related to the physiopathological status of their progenitor cells and can change in response to changes in physiological and pathological conditions [20, 30].

Exosomes are enriched in multiple proteins, both within and on their surface membranes. These proteins include adhesion molecules (e.g., integrins) [31], proteins responsible for membrane transport and fusion (e.g., annexins and Rab GTPases) [32], cytoskeletal proteins (e.g., actin and tubulin) [33], heat shock proteins (Hsps, e.g., Hsp70 and Hsp90) [34, 35], and proteins involved in the biogenesis of multivesicular bodies (MVBs), such as apoptosis-linked gene-2 interacting protein X (Alix) and tumor susceptibility gene 101 (Tsg101) [36]. Lysosomal proteins [e.g., lysosome-associated membrane glycoprotein 2b (Lamp2b)] [37] and surface tetraspanins (e.g., CD9, CD63, CD81, and CD82) [30, 38, 39] are also present in exosomes. The tetraspanins CD9 and CD81 facilitate direct membrane fusion between exosomes and target cells [40]. The tetraspanins CD55 and CD59 offer protection against complement membrane attacks [41]. Some of the above-mentioned proteins (CD9, CD63, CD81, Alix, Tsg101, and Hsp70) are often considered exosomal markers [30, 42]. Expression of the “self-marker” CD47 in some subsets of exosomes, a “don’t eat me” signal, avoids immune phagocytic clearance and increases the stability of exosomes in systemic circulation [43, 44]. Exosomes may also contain major histocompatibility complex (MHC) class I and II proteins that are responsible for antigen presentation [39].

The composition of exosomal phospholipid bilayers resembles that of their progenitor cells. The phospholipid bilayer is abundant in lipid rafts (submicroscopic membrane microdomains), which are rich in ceramides, cholesterol, sphingolipids, and phosphoglycerides. They are responsible for regulating cargo sorting into MVBs, exosome formation, rigidity, and structure [45, 46]. The lipid composition of exosomal membranes not only enables them to fuse directly with the plasma membranes of recipient cells, but also increases the physicochemical stability of exosomes in the extracellular environment [28]. This protects the exosomal cargo from degradation to ensure its integrity until it is distributed to target cells [18, 47].

In addition to their protein and lipid compositions, exosomes are carriers of a wide range of genetic materials that can be transmitted to neighboring and distant cells. These genetic materials include RNA molecules [e.g., messenger RNA (mRNA) and microRNA (miRNA)] and deoxyribonucleic acid (DNA) molecules (e.g., mitochondrial DNA and chromosomal DNA) [18, 47, 48]. Exosomal components and their main biofunctions are summarized in Table 1 [32, 34, 39, 40, 43, 44, 47–53].

**Table 1:** Common exosomal components and their main biofunctions

Exosomal component (category)	Class	Examples	Main biofunctions	References
Proteins	Proteins involved in exosome biogenesis	Alix; Tsg101	MVB biogenesis	[36]
	Adhesion molecules	Integrins-α and -β; P-selectin; lactadherin; ICAM-I	Cell anchorage, adhesion and uptake	[42, 45]
	Tetraspanins	CD9; CD63; CD81; CD82	CD9 and CD81: mediates direct fusion between exosomes and cell membranes of target cells CD63: exosomal cargo sorting	[49]
	Membrane transport and fusion proteins	Annexin; Flotillin; Rab GTPase	Responsible for membrane transport and fusion. Exosome secretion into the extracellular environment	[50]
	Cytoskeletal proteins	Actin; tubulin; myosin; cofilin	Cytosolic proteins involved in exosome formation and secretion	[42, 45]
	Heat shock proteins	Hsp70; Hsp90	Exosomes secretion and signaling processes	[34]
	Immune escaping proteins	“Self-marker” CD47; GPI-anchored CD55 and CD59	CD47: protection against macrophage clearance CD55 and CD59: protection against complement lysis	[41, 44, 51]
	Antigen-presenting proteins	MHC class I; MHC class II	Antigen presentation for immune activation	[52]
Lipids	Ceramides		Regulates cargo sorting into MVBs and exosome biogenesis	[45, 53]
	Cholesterol		Involved in exosome secretion	[45, 54]
	Sphingomyelin		Exosome structure and rigidity	[45, 55]
	Phosphatidylserine		Exosome biogenesis	[45, 46]
	Phosphatidylcholine		Exosome biogenesis and structure	[45, 46]
	Phosphatidylinositol		Exosome biogenesis and structure	[45, 46]
Nucleic acids	DNA fragments	Mitochondrial DNA and chromosomal DNA	The genetic material of exosomes can modulate the phenotype of the recipient cells, and influence various biological processes. They have been used as non-invasive tools for clinical diagnosis of various diseases	[45]
	Coding and non-coding RNAs	mRNA and miRNA		

*Alix* apoptosis-linked gene-2 interacting protein X, *DNA* deoxyribonucleic acid, *GPI* glycosylphosphatidylinositol, *Hsp* heat shock protein, *MHC* major histocompatibility complex, *miRNA* microRNA, *mRNA* messenger RNA, *MVB* multivesicular body, *RNA* ribonucleic acid, *Tsg101* tumor susceptibility gene 101, *ICAM-I* intercellular adhesion molecule-1

#### Exosome biogenesis

Exosome biogenesis typically involves: 1) invagination of the plasma membrane by inward budding, 2) accumulation of intraluminal vesicles within MVBs by inward budding of the MVB membrane, 3) fusion of MVBs with the plasma membrane, and 4) release of intraluminal vesicles as exosomes into the extracellular space upon fusion of the MVB with the plasma membrane [18, 47].

**Fig. 3:**
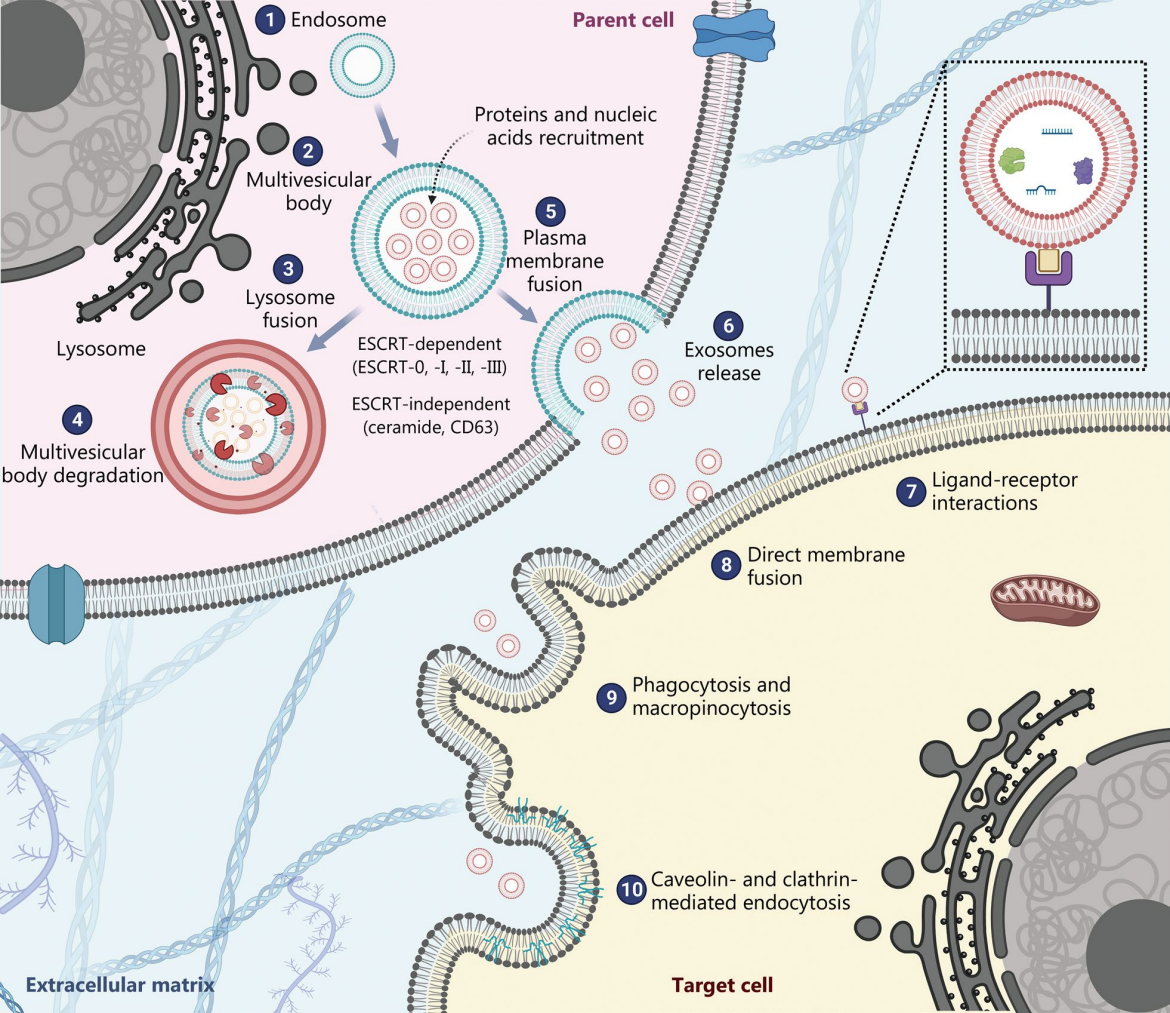
Exosome biogenesis and cell–cell communication. ① Biogenesis of exosomes begins with inward budding of the plasma membrane to form an early endosome. ② A second inward budding of the early endosome generates a multivesicular body containing intraluminal vesicles. During the second inward budding, exosomes are loaded with their cargo (mRNAs, non-coding RNAs, proteins, and DNA fragments). Exosomal biogenesis can occur through both ESCRT-dependent and ESCRT-independent pathways. ③ and ④ The multivesicular body can fuse either to a lysosome for the degradation of its components or to the plasma membrane for secretion. ⑤ and ⑥ The multivesicular body ultimately fuses with the plasma membrane to release its intraluminal vesicles into the extracellular space as exosomes. The released exosomes may be taken up by target cells through ⑦ receptor-mediated endocytosis, ⑧ direct fusion with the recipient plasma membrane, ⑨ phagocytosis and macropinocytosis, or ⑩ caveolin- and clathrin-mediated endocytosis. ESCRT endosomal sorting complex required for transport

Exosome formation begins with invagination of the plasma membrane by inward budding, forming early endosomes. These structures then undergo a sequence of alterations to form late endosomes, which are also known as MVBs, and which are characterized by the presence of several intraluminal vesicles in their luminal space. Intraluminal vesicles are formed by inward budding of the MVB membrane [30, 56]. Once MVBs containing several intraluminal vesicles are formed, they can have one of two different fates: 1) degradation by fusion of the MVB with a lysosome, or 2) exocytosis through fusion of the MVB with the plasma membrane, leading to the release of intraluminal vesicles as exosomes into the extracellular space (Fig. 3) [30, 38, 57]. Secretion of exosomes into the extracellular environment through exocytosis is dependent on soluble N-ethylmaleimide-sensitive fusion attachment protein receptors and Rab GTPases such as Rab-27a, RAB-11, and Rab-31 [38, 58, 59].

#### Mechanisms of exosome biogenesis

The most reported mechanism for the formation of intraluminal vesicles within MVBs involves the “endosomal sorting complex required for transport” (ESCRT). This complex comprises four protein complexes (ESCRT-0, ESCRT-I, ESCRT-II, and ESCRT-III) that function cooperatively to promote exosome biogenesis [18, 38, 47]. The ESCRT-dependent mechanism is initiated by the sequestration of ubiquitinated proteins by ESCRT-0, which subsequently recruits ESCRT-I and ESCRT-II. Both ESCRT-I and ESCRT-II are responsible for invagination of the MVB membrane. ESCRT-III causes the scission of inward budding vesicles [60], resulting in the formation of intraluminal vesicles [30].

Once the concomitant inhibition of all four ESCRT complexes has been shown not to suppress the formation of MVB, alternative ESCRT-independent mechanisms for MVB formation and exosome biogenesis have been suggested [61]. One of the proposed ESCRT-independent mechanisms is dependent on ceramides. A study conducted using mouse oligodendroglial cell lines showed that the secretion of exosomes did not require the ESCRT machinery, but was instead dependent on sphingomyelinase, an enzyme that catalyzes the production of ceramides [53]. The ESCRT-independent mechanism also appears to depend on the tetraspanin CD63, which is abundant in exosomes. It has been shown that CD63 plays an important role in mediating intraluminal vesicle formation [39, 62, 63].

### Cell–cell communication in physiological and pathophysiological processes

Initially, exosomes were perceived as a means by which cells discharged unwanted or unnecessary materials, and were thus regarded as cellular waste. Today, it is generally accepted that exosomes serve an additional function by communicating with proximal and distal cells to reprogram those cells [38, 43]. Cell–cell communication is crucial for homeostasis. Exosomes released by healthy and diseased cells function as important mediators of intercellular communication because previously enclosed biomolecules can be delivered to neighboring and distant cells [18, 26].

Once exosomes are released into the extracellular space, they are internalized by recipient cells, which then undergo phenotypic and behavioral changes. Three mechanisms have been proposed for the cellular internalization of exosomes: 1) direct fusion of exosomes with the cell membrane, 2) interaction with cell-surface receptors (ligand-receptor interactions), and 3) uptake of exosomes through endocytosis. The latter includes caveolin-mediated endocytosis, clathrin-mediated endocytosis, lipid-raft-mediated endocytosis, phagocytosis, and macropinocytosis (Fig. 3) [64, 65].

Exosomes provide an important mechanism for short- and long-distance cellular communication. They play a key role in physiological processes such as tissue repair, cell proliferation, blood coagulation, and immune surveillance [38]. Each exosomal source cell can impart specific biofunctionalities that can be employed when developing exosome-based therapies. Recent studies have reported an important role of exosomes in immunomodulation. Immune-cell-derived exosomes can trigger potent immune responses because of their antigen presentation capabilities. Because of the expression of MHC molecules on their surface, exosomes derived from B lymphocytes can present antigens to CD4^+^ and CD8^+^ T cells to induce strong immune responses [66]. Exosomes derived from T cells can retain the immunostimulatory and tumor growth inhibitory effects of their progenitor cells [67–70]. Exosomes secreted from macrophages are endowed with intrinsic tropism towards inflammatory and tumorous tissues [67, 71–74]. Mesenchymal stem cell (MSC)-derived exosomes can be derived from adipose tissue [75–77], bone marrow [78–81], umbilical cord [82, 83], and human placenta [84], and are most commonly used for tissue regeneration and wound-healing applications [76, 77, 85]. They also exhibit important immunomodulatory properties [86].

In addition to their physiological functions, exosomes also play a vital role in various pathological processes. Prior studies have documented the contribution of exosomes to the spread and progression of neurodegenerative [87], cardiovascular [88], and malignant diseases [89]. Tumor cell-derived exosomes exhibit properties that are similar to those of their parent cells. These exosomes transport tumor antigens to modulate the tumor microenvironment and facilitate tumor dissemination [90]. Exosomes derived from tumor cells are involved in tumor development, tumor cell proliferation, the generation of pre-metastatic niches, the promotion of tumor angiogenesis, and tumor immunosuppression [91–101]. This is achieved by suppressing the activity of natural killer cells, differentiating dendritic cells (DCs), and activating T lymphocytes [90]. Table 2 [65–83, 91–114] shows the exosomes source, biofunctionality and biomedical applications.

**Table 2:** Source of exosomes, biofunctionality and biomedical applications

Exosome source	Biofunctionality and biomedical applications	References
B cells	Immunomodulatory properties	[102]
	Stimulation of T-cells activation	[102]
T cells	Innate immune response modulation	[68–70]
	Tumor inhibition	[68–70]
Macrophages	Mediators in tumor progression, angiogenesis and metastasis formation	[71, 72]
	Inflammation targeting	[73]
	Cancer targeting	[67, 74]
MSCs	Tissue regeneration and tissue engineering	[103–107]
	Immunomodulatory properties	[108–110]
MSCs—adipose tissue	Immunomodulatory properties	[75]
	Reconstructive medicine and tissue engineering	[76, 77]
	Wound-healing	[85]
	Neurodegenerative disease remission	[111]
	Atherosclerosis management	[112]
MSCs—bone marrow	Cancer and metastasis targeting	[78]
	Tissue regeneration	[79, 80]
	Osteoarthritis reversal	[81]
MSCs—umbilical cord	Chondrogenic effect	[82]
	Tissue repair	[83]
MSCs—human placenta	Tissue restauration after acute ischemic stroke	[84]
Tumor cells	Targeting to tumor cells	[93]
	Immunomodulatory activities	[94]
	Mediators in tumor progression, angiogenesis and metastasis formation	[95–98]
	Natural source of tumor-specific antigens	[99–101]
Endothelial cells	Cardioprotective effects	[113]
	Endothelial dysfunction reversal	[114]
Neural stem cells	Neuroprotective effects	[115]
	Cancer cells growth inhibition	[116]

*MSCs* mesenchymal stem cells

## Exosomes versus liposomes as drug delivery systems: a comparative overview

Liposomes and exosomes, biological and highly complex liposomal forms, are remarkably similar in terms of diameter and phospholipid bilayer structure, which resembles that of cell membranes [117, 118]. A distinctive feature of exosomes is their complex surface repertoire, which is responsible for enhancing cell-specific targeting and uptake [118–120]. Both of these amphiphilic vesicles are promising delivery mechanisms for both hydrophobic and hydrophilic drugs [18, 121].

Liposomes are lipid-based drug delivery systems of a synthetic nature with well-documented therapeutic benefits [121]. However, concerns related to the lack of specific-cell targeting, inability to cross biological barriers, rapid elimination from blood circulation, and immunogenicity have triggered the search for more “biologically inert” approaches [117, 122–124].

The idea of harnessing exosomes as drug delivery systems stems from the role natural exosomes play in intercellular communication. Naturally occurring exosomes have emerged as a more complex and biocompatible alternative to liposomes for drug delivery [119]. Some attributes of exosomes that make them more ideal than liposomes for drug delivery include enhanced biocompatibility, non-immunogenicity, and intrinsic cell-specific targeting. The latter is ascribed to the ability of exosomes to preserve the surface membrane composition and intrinsic targeting properties of their progenitor cells [19, 125, 126]. Exosomes secreted by specific cell types exhibit intrinsic cell tropism, which favors their uptake by target cells via well-established mechanisms [44, 119, 120, 127, 128]. Another advantage of natural exosomes as drug delivery systems is their optimal nanoscale size, which facilitates their penetration through biological barriers, such as the blood–brain barrier [129]. In addition, some subsets of exosomes are capable of evading immune recognition and clearance owing to the presence of the “self-marker” CD47 on their surface [130]. Evasion of immune surveillance increases exosomes’ duration of systemic circulation and protects their cargo from degradation [18, 44, 126]. Natural exosomes and synthetic liposomes as advanced drug delivery systems are shown in Table 3 [16, 117–122, 125–127, 129–132].

**Table 3:** Natural exosomes versus synthetic liposomes as advanced drug delivery systems

Properties	Exosome	Liposome	References
Structure	Naturally enriched with lipids, proteins, and nucleic acids	Composed of lipids, but no proteins and nucleic acids are present	[119]
Origin	Biological origin (naturally released by cells)	Synthetic origin (bottom-up approach)	[119, 120]
Complexity of contents	Heterogeneous composition (low control of contents)	Homogeneous composition (high control of contents)	[119, 120]
Polydispersity	Polydisperse	Monodisperse	[120]
Drug loading capacity	Low loading efficiency; both hydrophobic and hydrophilic drugs can be loaded	High loading efficiency; both hydrophobic and hydrophilic drugs can be loaded	[18, 121]
Immunogenicity	Absent (high biocompatibility)	Shows immunogenicity	[120, 122–124]
Targeting features	Natural organotropism (ascribed to binding proteins expressed on surface membrane)	Low organotropism *per se* (surface ligands must be added for improving cell targeting)	[119, 120, 127]
Cell internalization	Cell uptake occurs via several well-established mechanisms	Cell uptake occurs via non-established mechanisms	[120, 128, 131]
Ability to cross biological barriers	Present	Absent	[129]
Systemic half-life	Short half-life (approximately 60 min after administration*)	Reduced half-life (incorporation of PEG can confer stealth features)	[132, 133]
Industrial scale production	Very challenging (clinical-scale production methods are missing)	Easy clinical-scale manufacturing	[120, 134]

*PEG* polyethylene glycol

*Although it has been suggested that exosomes are rapidly cleared from bloodstream after administration, studies are reporting that blood circulation of CD47-expressing exosomes can be substantially improved

A close comparison of the biodistribution and pharmacokinetic profiles of liposomes and exosomes is unfortunately very limited and controversial [119]. A great deal of evidence has suggested that natural exosomes are rapidly eliminated from the bloodstream [135, 136], and similar to liposomes, they suffer non-specific accumulation in the liver [137–139]. Despite these comparable clearance rates [117], the pharmacokinetic benefits of some subsets of exosomes over liposomes have been strongly supported, with one study showing superior blood circulation of exosomes when compared to liposomes [132]. Bloodstream exosomes were detected 24 h after administration in vivo, which was ascribed to the privileged immunological features of exosomes conferred by innately surface-expressed CD47 [132]. Thus, the in vivo pharmacokinetics of exosomes appear to be related to their membrane protein profiles [133, 139].

In conclusion, when compared to liposomes, exosomes surface-enriched in CD47 can substantially reduce immune clearance; however, further evidence is required. There is still much to be discovered regarding the in vivo fate of exosomes, and the extent to which CD47 expression can shield exosomes from immune recognition and clearance should be further investigated [117]. Despite the site-specific targeting and pharmacokinetic superiority of natural exosomes over liposomes, the complexity and heterogeneity of intra-exosomal contents and the low production and isolation yields of exosomes remain challenging issues for clinical translation [120, 134].

## Types of exosomes

Current exosome-based therapeutic platforms include natural exosomes and artificial exosomes (exosome-like NPs). Natural exosomes are endogenous cell-secreted nanovesicles that carry functional biomolecules from their progenitor cells. In addition to their endogenous cargo, exogenous therapeutics can also be loaded into naturally occurring exosomes either by modifying exosome progenitor cells (transfection of progenitor cells) or by loading exosomes directly with specific cargo [20]. Artificial exosomes are synthetic counterparts engineered to possess superior biopharmaceutical acceptability.

### Natural exosomes

Natural exosomes have attracted considerable attention owing to their potential diagnostic and therapeutic applications. The physiopathological status of their progenitor cells has a significant impact on the cargo content of natural exosomes, highlighting the interest in using exosomes as biomarkers for pathological conditions [140]. Exosomes can be found in biological fluids such as blood [141], saliva [142], urine [143], and ascites [144], and have been used to non-invasively diagnose a wide range of human diseases [19, 145].

### Exosomes carrying exogenous therapeutic cargo

Exosomes have been used experimentally as nanocarriers to deliver various therapeutic cargoes, such as anti-cancer drugs [146, 147], therapeutic proteins [148], nucleic acids [149, 150], and nanomaterials [151], for the treatment of various human diseases. These include cardiovascular and neurodegenerative diseases, wound healing, and cancer applications, with the latter being one of the most researched areas in exosomal therapy—the loading of exosomes with chemotherapeutic molecules has received considerable attention [152]. Exosomes were engineered to express surface ligands that could bind to specific molecules overexpressed by tumor cells to achieve greater accumulation at tumor sites. To further enhance the ability of exosomes to actively target tumor sites, immature DCs were genetically modified to express Lamp2b, an exosomal surface protein that interacts with αV integrin overexpressed by tumor cells [152]. Engineered doxorubicin-loaded exosomes enabled more efficient delivery of doxorubicin to breast cancer cells (95.4%), which was significantly higher than that of non-engineered doxorubicin-loaded exosomes (35.0%). This resulted in improved antitumor performance in vivo [152].

Exosome-based platforms have also been shown to be effective tools for neurodegenerative disease therapy, such as for Parkinson’s disease (PD) [153] and Alzheimer’s disease (AD) [154, 155]. As already mentioned, exosomes can penetrate the blood–brain barrier for effective brain-targeted drug delivery. For instance, both in vitro and in vivo experiments have shown that dopamine-loaded blood exosomes can effectively cross the blood–brain barrier for the targeted delivery of dopamine to the brain. Dopamine-loaded exosomes not only increased drug bioaccumulation in neuronal cells by more than 15-fold, but also reduced systemic toxicity and improved therapeutic efficacy against PD [153]. Regarding therapies targeting AD, several compounds have been encapsulated in natural exosomes, namely curcumin [154] and quercetin [155]. Exosomal drug loading resulted in 2.5-fold-higher brain accumulation in vivo when compared to free quercetin [155]. Similarly, an in vitro study has demonstrated the superior blood–brain barrier crossing ability of curcumin-loaded exosomes when compared to free drugs (60% and 15%, respectively) [154]. Both exosome-based platforms significantly improve cognitive dysfunction and alleviate AD symptoms by suppressing tau protein phosphorylation, thus showing promising results for AD therapy [154, 155].

Exosomal therapy has also recently been applied in wound-healing applications [156]. MSCs have attracted considerable interest for their ability to accelerate wound healing by stimulating cell proliferation and angiogenesis. MSC-derived exosomes have emerged as a novel, cell-free strategy for wound-healing applications because of their progenitor cell-related, tissue-regenerating properties. For instance, in a recent study, a miR-155 inhibitor was loaded into natural exosomes to yield an exosome-based system with synergistic effects on diabetic wound healing and closure [156]. The superior diabetic wound-healing effects of the loaded exosomes were clearly demonstrated in vitro and in vivo, yielding enhanced collagen deposition, re-epithelialization, and angiogenesis [156].

### Artificial exosomes: biomimetic exosome-like nanomaterials

**Fig. 4:**
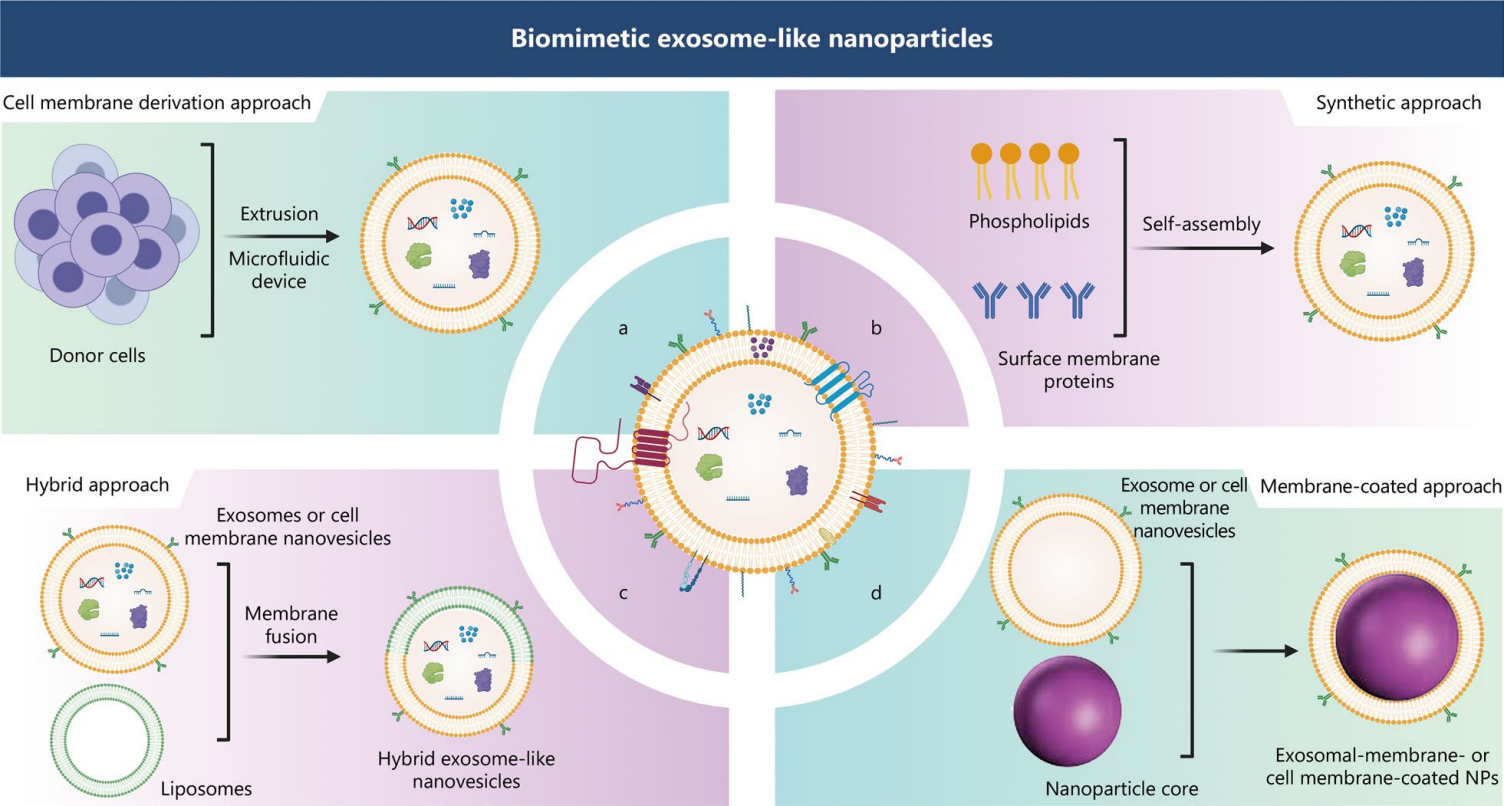
Approaches to constructing biomimetic exosome-like nanoparticles. Approaches to constructing biomimetic exosome-like NPs, which can be used as an alternative to natural exosomes, include: a generation of exosome-mimetic nanovesicles through direct extrusion of progenitor cells through porous membranes or by forcing them to move through microfluidic devices (**a**, a top-down approach). Preparation of synthetic exosome-like NPs using a synthetic strategy inspired by natural exosomes that involves self-assembly of synthetic phospholipid bilayers with specific antibodies, peptides, or surface proteins from biomembranes (**b**, a bottom-up approach). Fabrication of hybrid exosome-like nanovesicles by fusion of nanovesicles (natural exosomes or cell membrane nanovesicles) with synthetic liposomes through a top-down approach (**c**), or by fusion of two or more different biomembranes to generate hybrid membranes that incorporate multiple functionalities of different membrane types, and generation of exosomal-membrane- or cell membrane-coated NPs by coating NP cores with biomembranes (exosomal membranes or cell membrane nanovesicles, respectively) via top-down approaches (**d**). NP nanoparticle

Despite the promising potential of naturally cell-secreted exosomes as drug delivery systems, their clinical use is hindered by the reduced number of exosomes naturally secreted by most cells, poor production and isolation yields, a lack of standardized methods for exosome isolation and purification, and low encapsulation efficiency [18, 157]. To overcome these limitations, extensive efforts have been devoted to the study of bio-inspired exosome-like NPs (Fig. 4). These artificial exosomes include: 1) exosome-mimetic nanovesicles, 2) synthetic exosome-like NPs, 3) hybrid exosome-like nanovesicles, and 4) exosomal membrane-coated NPs. A comparative analysis of natural exosomes and different strategies used to yield artificial exosomes is presented in Table 4 [156–166].

**Table 4:** Comparison between natural exosomes and the different strategies used to yield artificial exosomes

Type	Source	Biomimetic profile	Production quantity	Production complexity	Programmability/tunability	References
Natural exosomes	Naturally released from parent cells (isolated from the cell supernatant)	+++	‒	++	+	[158, 159]
Artificial exosomes (exosome-like NPs)	Extrusion of donor cells through porous membranes or forcing them to move through microfluidic devices (Top-down strategy)	++	+++	+	++	[158, 160–163]
	Self-assembly of synthetic phospholipid bilayers (liposomes) with antibodies, peptides or membrane proteins (Bottom-up strategy)	+	++	+	++	[158, 164]
	Fusion of two nanovesicles of different origins to yield hybrid structures (Top-down strategy)	–	++	++	++	[158, 165, 166]
	Coating of NP cores with biomembranes (exosomal membranes or cell membrane nanovesicles) (Top-down strategy)	++	++	+	++	[167, 168]

– Indicates that the parameter is reduced; + indicates that the parameter is slightly elevated; ++ indicates that the parameter is moderately elevated; +++ indicates that the parameter is extremely elevated. *NP* nanoparticle

#### Cell membrane derivation approach

Scalability remains a significant challenge for the clinical application of natural exosomes. The preparation of exosome-mimetic nanovesicles by the direct disassembly of progenitor cells through top-down approaches (i.e., the disintegration of complex and large molecules into less complex and smaller units) is an effective approach for the stable and scalable production of artificial exosomes. These artificial exosomes help address the low production yield of natural exosomes [158, 160]. This strategy involves the direct extrusion of progenitor cells through porous membranes, and is the most commonly used approach [162, 169, 170]. Alternatively, artificial exosomes can also be produced by forcing cells to move through the microchannels of microfluidic devices [171, 172] (Fig. 4a). The resulting cell-derived nanovesicles have the membrane surface composition and intrinsic targeting features of natural exosomes. For example, doxorubicin-loaded exosome-mimetic nanovesicles have been formed by extruding doxorubicin-loaded monocytes/macrophages through membrane filters. The resulting exosome-mimetic nanovesicles were similar in size and morphology and contained surface protein markers similar to those of natural exosomes. The production yield of nanovesicles was 100-fold higher than that of exosomes. After in vivo administration, exosome-mimetic nanovesicles accumulated efficiently in tumor tissues and inhibited tumor growth [173].

Applications for exosome-mimetic nanovesicles in wound-healing [174] and regenerative medicine [175] have also been reported. Recently, human umbilical MSCs have been repeatedly extruded through porous membranes to generate MSC-derived exosome-mimetic nanovesicles. These nanovesicles were more effective than MSC-derived exosomes in promoting wound-healing by stimulating dermal fibroblast proliferation [174]. Hepatocyte-derived exosomes play a prominent role in liver regeneration [175]. To address the low production yield of natural exosomes, hepatocytes were extruded through porous membranes to produce exosome-mimetic nanovesicles with a 100-fold higher production yield than exosomes. The resulting nanovesicles effectively stimulated liver cell proliferation and regeneration [175].

#### Synthetic approach

The preparation of synthetic exosome-like NPs using bottom-up approaches has been used to address the heterogeneity and safety concerns of natural exosomes. These bottom-up approaches involve building large and complex molecules by assembling small and less-complex units [158]. Synthetic exosome-like NPs are synthetic constructs inspired by natural exosomes (Fig. 4b). These NPs only include the essential components of natural exosomes. Their preparation involves the assembly of synthetic phospholipid bilayers (e.g., liposomes) that mimic the lipid composition and size of natural exosomes [176, 177]. For instance, exosome-like liposomes (with a lipid composition mimicking that of natural exosomes) were produced and efficiently used as carriers of curcumin for AD therapy, with an encapsulation efficiency of 94% [176]. These biomimetic exosome-like NPs increase curcumin stability and brain distribution, enhancing its neuroprotective effects against AD-related oxidative stress [176]. In addition, the assembled phospholipid bilayers can be subsequently functionalized with specific antibodies [178], peptides [179], and proteins [180], or coupled with membrane proteins extracted from cell membranes [164]. For example, leukocyte-mimicking liposomes (leukosomes) have been designed by incorporating membrane proteins extracted from leukocytes into synthetic liposomes. Leukosomes showed ninefold greater accumulation at melanoma sites than liposomes, meaning that they delivered doxorubicin more efficiently. This resulted in a more targeted therapy with superior antitumor efficacy [181]. In another study, proteins extracted from cancer-cell membranes were incorporated into synthetic liposomes to yield biomimetic liposomes for triple-negative breast cancer therapy. These biomimetic liposomes were coupled with surface-bound elastase to destroy the tumor extracellular matrix and facilitate drug and cytotoxic T cell infiltration. Elastase-bound biomimetic liposomes showed tumor-targeting capability, fostering the accumulation of chemotherapeutics at tumor sites [182].

Apart from modifying liposomes with membrane proteins extracted from cell membranes, surface proteins can also be incorporated into phospholipid bilayers using a cell-free protein synthesis technique [180]. For example, connexin 43 (Cx43)-embedded liposome-coated chitosan NPs have been synthesized using exosome-mimicking phospholipid bilayers. Exosome-like liposomes were used to deliver small interfering RNAs (siRNAs) targeting vascular endothelial growth factor (VEGF) to glioblastoma cells [180]. The chitosan NPs were first loaded with VEGF siRNA through electrostatic interactions and subsequently camouflaged with exosome-mimicking membranes. Cx43 integration improved glioblastoma cell delivery efficiency via Cx43-mediated gap-function channels, resulting in a 30% reduction in VEGF expression [180]. In a similar effort to enhance the cell internalization efficiency of liposomes, exosome-mimicking liposomes were created to combine the advantages of both entities [131]. The cell uptake efficiency of exosomes was 3-fold higher than that of liposomes due to the exosomes’ leveraging of cell internalization mechanisms [131].

#### Hybrid approach

Hybrid exosome-like nanovesicles have been prepared using top-down approaches to combine the biological functions of natural exosomes with the pharmaceutical benefits of nanomaterials [158]. The fabrication of hybrid exosome-like nanovesicles involves the fusion of the membranes of nanovesicles (natural exosomes or cell membrane nanovesicles) with synthetic liposomes (Fig. 4c), thus combining the benefits of exosomes and liposomes [165, 183, 184]. For example, exosome-liposome hybrids were prepared through membrane fusion of Raw264.7 cell-derived exosomes with synthetic liposomes using a freeze–thaw method. The cell internalization efficiency of Raw264.7 cell-derived exosome-liposome hybrids was almost 2-fold higher than that of natural exosomes [185].

Another variation of the hybrid approach involves the fusion of two or more biomembranes to create a hybrid membrane that incorporates the functionalities of each membrane. One of these hybrid systems was produced by fusing platelet membranes with membranes of bone marrow MSC-derived EVs for the treatment of ischemic heart disease. This hybrid system combines the intrinsic injured vasculature-targeting ability of platelets with the pro-angiogenic functions of EVs. The hybrid nanovesicles showed 1.8-fold higher accumulation in ischemic heart areas than unmodified EVs [186]. In another study, MSC-derived exosomes were fused with the platelet membrane via extrusion to yield a hybrid system for the treatment of myocardial infarction (MI). The hybrid nanovesicles were readily taken up by endothelial cells and cardiomyocytes because of their inherent ability to target injured vasculature, resulting in improved cardiac function in vivo [187].

#### Membrane-coated approach

**Fig. 5:**
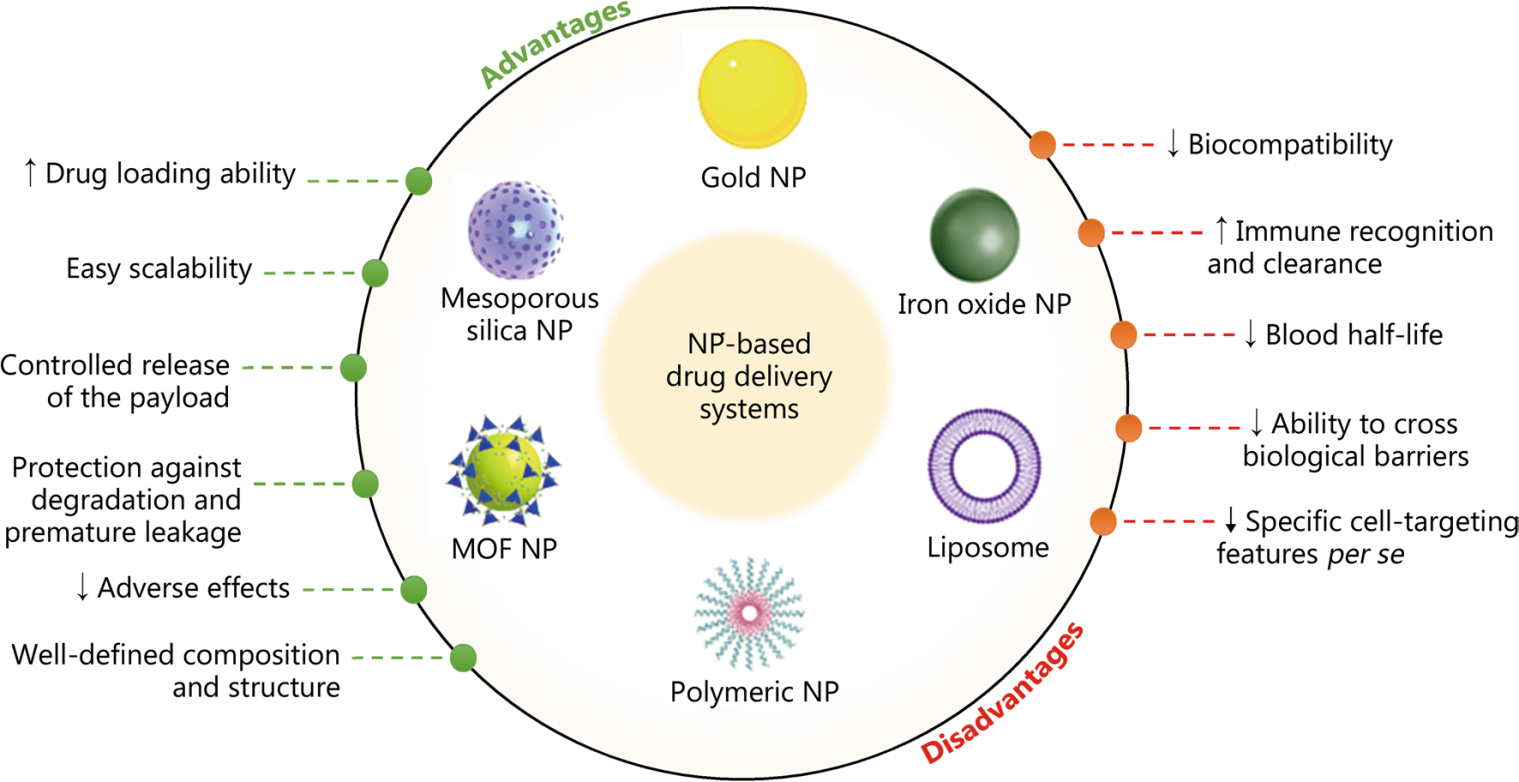
Depiction of the main advantages (left) and disadvantages (right) of current nanotechnology-based drug delivery systems. ↑ indicates enhancement, ↓ indicates reduction. MOF metal organic framework, NP nanoparticle

Nanoscale materials are nanotechnological tools that are well-suited for drug delivery. Nanotechnology-based drug delivery systems can enhance the therapeutic and safety goals of conventional therapies, improving the diagnosis and treatment of various human diseases. However, despite the promising potential of nanomaterials as drug delivery systems, some drawbacks hinder their clinical translation [188] (Fig. 5).

To overcome some of the aforementioned drawbacks of nanoscale materials, recent studies have focused on coating nanomaterials with various types of biological membranes to produce biomimetic carriers. This helps to improve the interfacial properties of NPs, endowing them with prolonged systemic circulation and enhanced biocompatibility, immune evasion, and tissue specificity [12]. The membranes used for coating have included natural cell membranes and subcellular structures, such as membranes derived from exosomes (Fig. 4d).

Exosomal-membrane-coated NPs combine the advantages of endogenous exosomes (enhanced biocompatibility, reduced clearance by the mononuclear phagocyte system, and tissue specificity) with the pharmaceutical benefits of nanomaterials (higher drug-loading ability, easy scalability, greater flexibility to undergo surface modification, and controlled drug release) while overcoming their limitations [18, 19]. Exosomal-membrane-coated NPs are generated by coating the inner core of an NP with an exosomal membrane using a top-down approach. Thus, the inherent biological features of the exosomal membrane can be preserved and transferred to the NP [18, 19]. Hence, surface-engineering via exosomal-membrane-coated nanosystems offers substantial benefits over non-coated nanomaterials by extending their systemic half-life and enhancing tissue specificity [189]. For instance, the uptake of exosomal-membrane-coated metal–organic framework NPs by macrophages was reported to be only 30% of that of uncoated NPs [190]. In another study, exosomal membrane functionalization improved targeted accumulation in homotypic murine 4T1 breast tumors by 3.1-fold when compared to their non-coated counterparts [191].

## Fabrication: engineering of exosomal-membrane-coated NPs

**Fig. 6:**
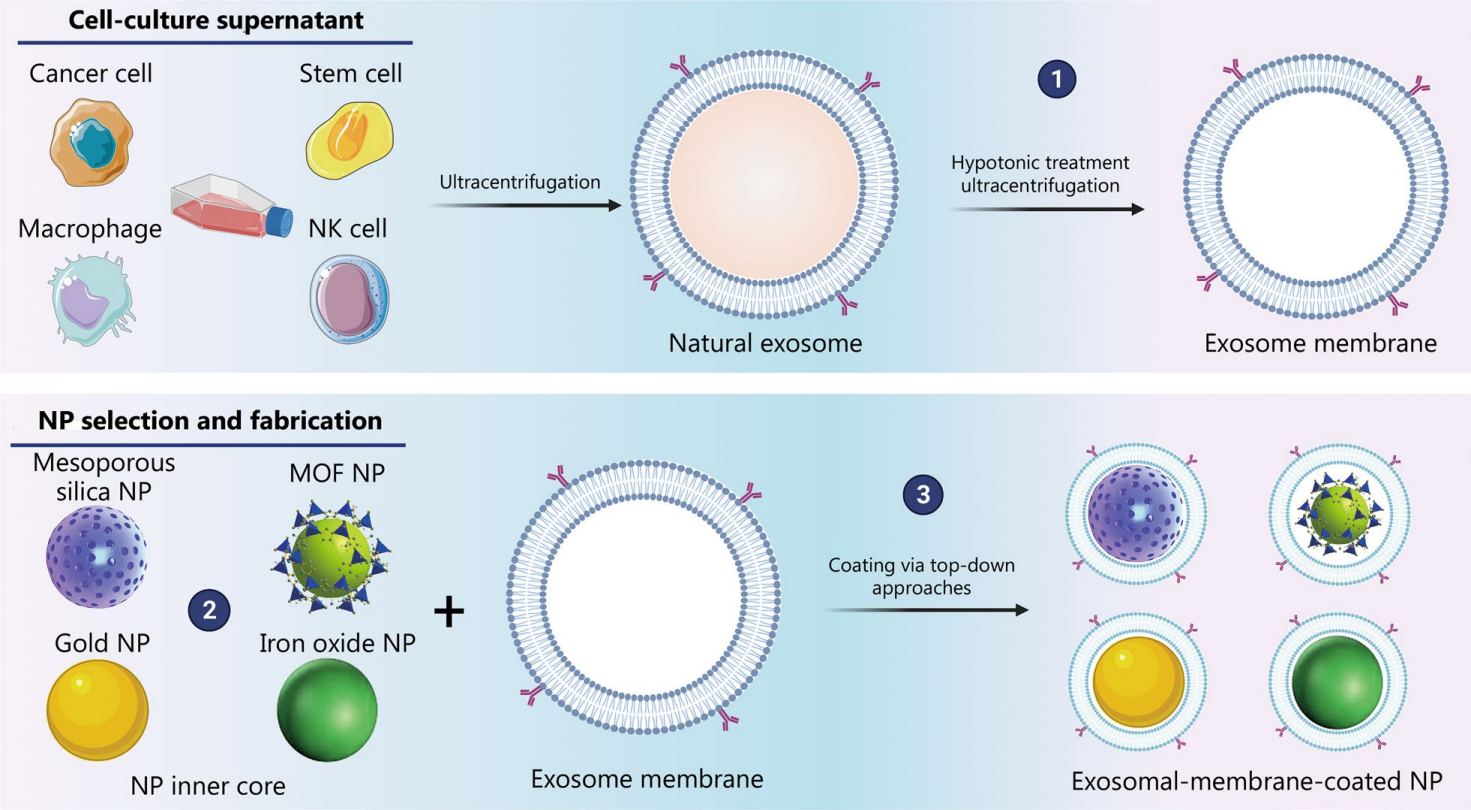
Three steps involved in synthesizing exosomal-membrane-coated nanosystems. ① extraction of exosomal membranes through hypotonic treatment of exosomes that were previously isolated from cell-culture supernatants via ultracentrifugation, ② selection and fabrication of the NP inner core, and ③ coating of the synthesized NP inner core with the extracted exosomal membrane via top-down approaches to obtain a core–shell nanostructure. MOF metal organic framework, NP nanoparticle, NK natural killer

As shown in Fig. 6, the preparation of exosomal-membrane-coated nanosystems typically comprises three steps: 1) extraction of exosomal membranes through hypotonic treatment of exosomes, 2) selection and synthesis of the NP inner core, and 3) coating of the synthesized NP core with the extracted exosomal membrane to form a core–shell nanostructure [192].

### Exosomal membrane extraction

The preparation of exosomal-membrane-coated NPs requires the extraction of the membrane through hypotonic treatment of exosomes. This treatment removes intravesicular components while leaving the surface membrane proteins intact. Surface membrane proteins play important roles in cell recognition, signaling, and communication [125].

Natural exosomes were collected from the cell-culture supernatant via ultracentrifugation (differential centrifugation). In line with MISEV guidelines (2015), this is the most commonly used and reliable method for isolating exosomes from cell-culture supernatants [22, 38]. Several techniques have been proposed. However, there are currently no standardized methods for exosome isolation. Once isolated, exosomes should be analyzed and characterized. According to MISEV guidelines, several techniques must be employed for the characterization of isolated exosomes. These include transmission electron microscopy to analyze surface morphology, NP-tracking analysis for size, and Western blotting for the detection of exosomal surface proteins [22]. The exosomal membranes are then extracted by resuspending the collected exosome pellets in a hypotonic lysis buffer containing a protease inhibitor cocktail. The lysate is then ultracentrifuged to remove intravesicular contents and isolate the exosomal membrane. Finally, the membrane-rich fraction is washed with isotonic buffers, such as phosphate-buffered saline, to collect purified exosomal membranes [190, 193].

### Nanoparticle inner core selection and synthesis

The next step involves the selection and preparation of the NP inner core. Different nanomaterials have been used, ranging from organic cores to inorganic cores. Regardless of the NP composition and cell membrane types, it is essential to ensure that the nano-sized inner core has a negative zeta potential to facilitate electrostatic repulsion between the negatively charged NP surface and negatively charged membrane components [194]. This facilitates the correct orientation of the exosomal membrane around the NP core [195]. Cationic NP cores may hamper the coating process, as strong electrostatic interactions can lead to the unwanted bridging of the membrane structures and NP core materials, as described previously [196]. Other relevant parameters related to the NP core and NP core-membrane interfacial interactions that warrant further investigation are the NP’s core size, the surface curvature of the phospholipid bilayer, the impact on the sidedness of the membranes, and the completeness of the membrane coating.

Both organic and inorganic NP cores have been explored for the assembly of exosomal-membrane-coated NPs. The natural physicochemical properties of nanomaterial cores are related to their functionality. For instance, organic NPs are known for their biocompatibility, biodegradability, and high drug-loading capacity, and have mostly been employed in drug and gene delivery approaches. Liposomes [189] and poly(lactic-*co*-glycolic acid) (PLGA) NPs [197] have been used as NP cores and further coated with exosomal membranes. Poly(caprolactone) and human serum albumin NPs have also been used in this way [198]. In addition, inorganic-based nanomaterials such as mesoporous silica NPs [191], gold NPs (Au NPs) [199, 200], and iron oxide NPs [199] have been investigated for this purpose. Despite the bottlenecks associated with a lack of biodegradability, reduced biocompatibility, and toxicity when compared to organic-based nanoplatforms, inorganic NP cores have interesting properties for use in exosomal-membrane-coating approaches. The most commonly used cores are metallic NPs that exhibit intrinsic photothermal activity [191, 201]. Other strategies using metallic NP cores enable unique imaging features in exosomal-membrane-coated systems [199, 200], as well as magnetic properties for magnetic guidance-enhanced targeted migration [202]. The overall exosomal-membrane coating of inorganic NP cores is associated with a further increase in the biocompatibility of the nanosystem and introduces a more facile and tunable method for surface functionalization, as the traditional processes for ligand attachment onto the surface of inorganic NPs can be complex and system-specific.

### Coating the nanoparticle core with an exosomal membrane

Extracted exosomal membranes can then be used to camouflage the NP core. This can be achieved using different coating methods similar to those used for camouflaging NPs with natural cell membranes [21]. So far, different strategies have been reported for assembling exosomal-membrane-coated NPs. These include physical extrusion through porous membranes, sonication, direct incubation of NPs with living cells, direct incubation of NPs with isolated cell-secreted exosomes, as well as microfluidic sonication-based techniques.

#### Co-extrusion/sonication

Co-extrusion through porous membranes followed by sonication is the most extensively used approach for assembling exosomal-membrane-coated NPs. Physical extrusion, also known as co-extrusion, was the first reported coating method, and is commonly used to prepare synthetic liposomes. In this method, the NP inner core and purified exosomal membrane are combined and co-extruded through porous membranes to produce exosomal-membrane-coated NPs [197, 199, 203]. The disruptive mechanical forces induced by physical extrusion can disrupt the exosomal membrane’s structure, enabling it to reassemble around the NP surface to form a core–shell nanostructure [197, 199, 203]. Another approach used to coat the NP core with the exosomal membrane is sonication. In this approach, both the NP and the purified exosomal membrane are exposed to similarly disruptive forces that are generated by ultrasonic energy, resulting in the spontaneous formation of a core–shell nanostructure [201, 204]. This approach has the advantage of losing less material when compared to physical extrusion [205, 206].

#### Direct incubation of NPs with cells or exosomes

Although physical extrusion and sonication are widely used to camouflage NPs with exosomal membranes, these approaches are labor-intensive and time-consuming. There is also the possibility of damaging the protein integrity of exosomal membranes using these techniques [18]. Because surface membrane proteins are critical for the biological functions of exosomes, damaging their integrity adversely affects the biological properties of these biomimetic nanosystems [207]. To prevent damage, non-disruptive coating techniques have been adopted to coat NPs with exosomal membranes. One of these approaches is based on the direct incubation of NPs with living cells to enable cells to secrete NP-containing exosomes. This strategy takes advantage of the exosome biogenesis pathway to encapsulate NPs in the exosomal membrane [200]. In another approach, exosomal-membrane-coated NPs are produced by direct incubation of NPs with pre-collected exosomes [208, 209].

#### Microfluidic sonication method

To overcome the limitations of physical extrusion and sonication, a microfluidic sonication-based coating technique was recently proposed for the design of core–shell PLGA NPs in a single continuous manner. This technique utilizes ultrasonication to coat PLGA NPs with several types of biological membranes, including lipid, exosomal, and cancer-cell membranes [44]. The membranes of the exosomes and cancer cells were isolated from A549 human lung carcinoma cells. They were used to coat PLGA NPs using a microfluidic sonication approach. The exosomal-membrane-coated PLGA NPs showed 1.0- and 5.5-fold-higher accumulation at A549 tumor sites when compared to cancer-cell-membrane- and lipid-membrane-coated NPs, respectively. These improved results were attributed to the homotypic targeting ability of exosomes and reduced immune uptake by monocytes/macrophages [44].

The microfluidic sonication approach was used in a subsequent study for coating PLGA NPs with MDA-MB-231 cell (an epithelial, human breast cancer cell line)-derived exosomal membranes that were functionalized with AS1411 aptamers [210]. Because of the exosomal membrane coating, the biomimetic nanosystem exhibited a systemic circulation duration that was 3.5-fold longer than that of AS1411-modified lipid-PLGA NPs. In addition, owing to the specific binding of AS1411 aptamers to nucleolin, a nucleolar protein that is overexpressed on the membrane of some cancer cells, the NPs demonstrated 1.59-fold-higher accumulation in tumors when compared to exosomal-membrane-coated NPs without AS1411 functionalization [210].

## Biomedical applications in tissue engineering and neurodegenerative diseases

**Fig. 7:**
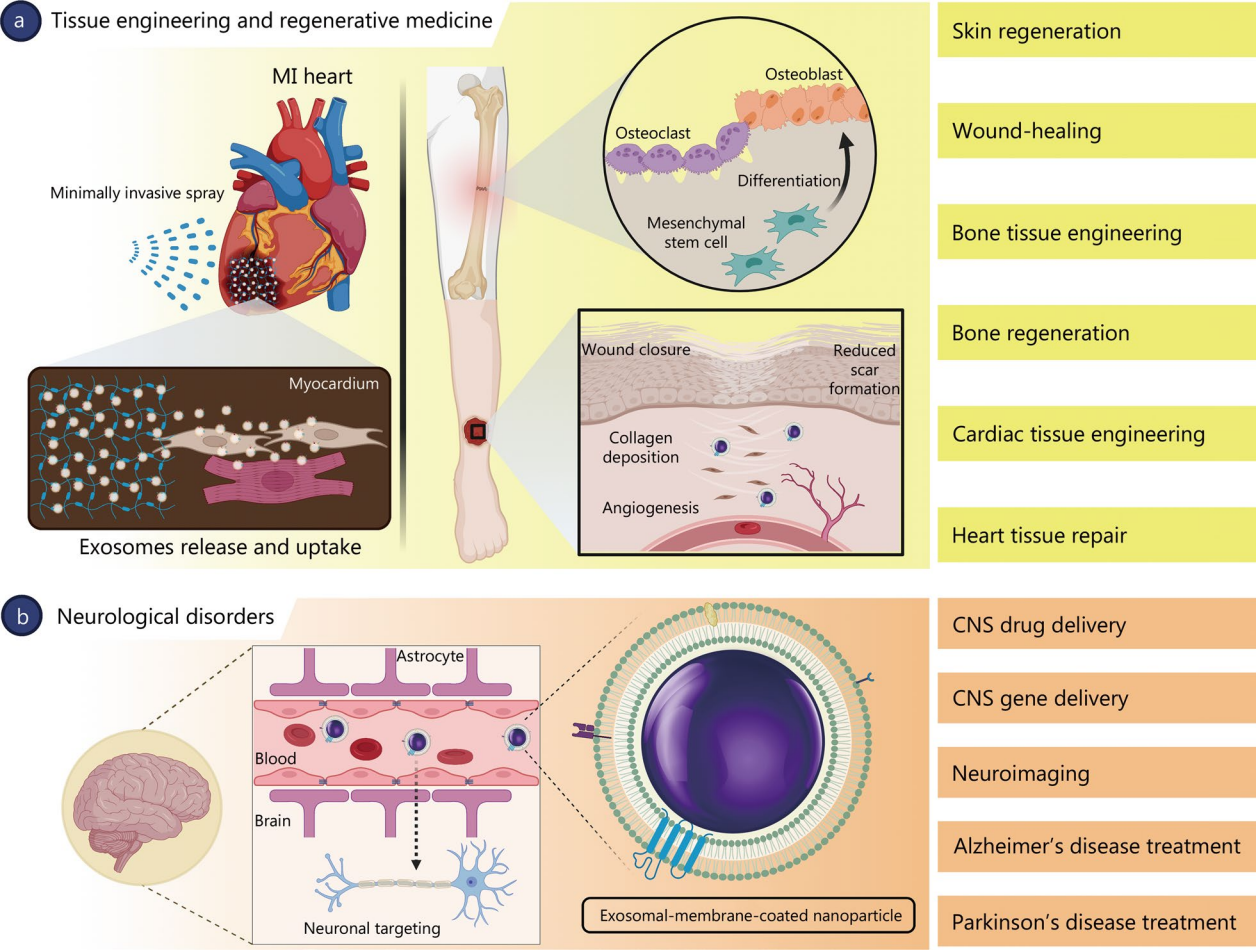
Biomedical applications of exosomal-membrane-coated nanosystems. **a** Tissue engineering and regenerative medicine (e.g., skin regeneration and wound-healing applications). **b** Neurological disorders (e.g., neuroimaging and treatment of Alzheimer’s disease and Parkinson’s disease). CNS central nervous system, MI myocardial infarction

The following sections highlight some studies that employ exosomal-membrane-coated nanosystems for biomedical applications. Figure 7 summarizes the biomedical applications of exosomal-membrane-coated NPs for tissue engineering and regenerative medicine, as well as the diagnosis and treatment of neurodegenerative diseases.

### Tissue engineering and regenerative medicine

The purpose of tissue engineering and regenerative medicine is to generate viable human tissues and organs to replace diseased or damaged ones or to induce their regeneration in vivo [211, 212].

MSCs are multipotent cells that are promising for treating inflammatory diseases and cutaneous wounds owing to their multipotent differentiation and immunosuppressive and regenerative properties [18]. The therapeutic effects of MSCs on skin regeneration and wound-healing appear to be related to their ability to promote angiogenesis, enhance collagen synthesis and re-epithelialization, and accelerate skin regeneration and wound closure. Recently, MSC-derived exosomes have been investigated for skin regeneration and wound-healing as they can maintain the functional properties of their progenitor cells [202].

**Fig. 8:**
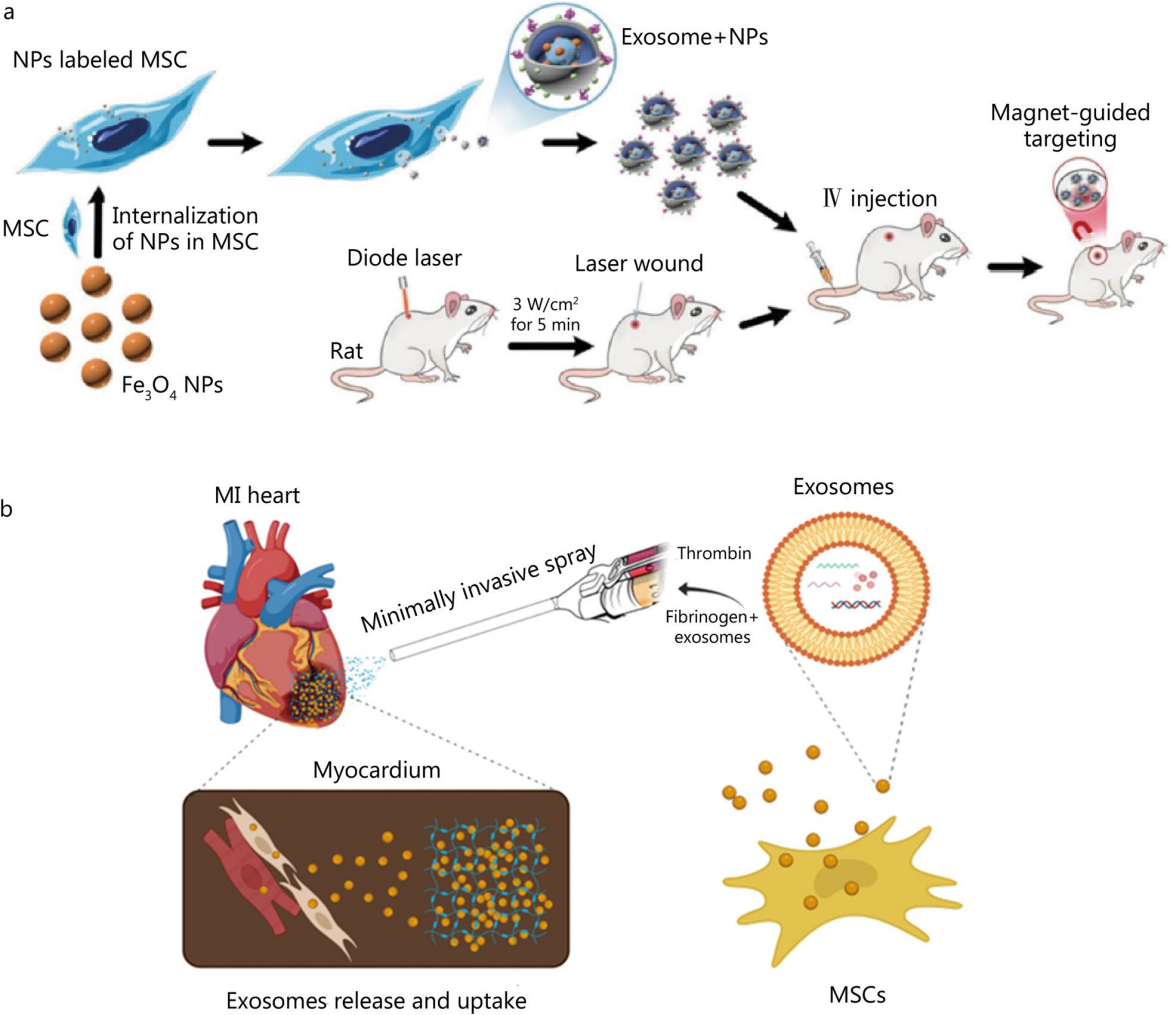
Exosome-based nanosystems for tissue engineering and regenerative medicine applications. **a** Preparation of MSC-derived exosomal-membrane-coated Fe_3_O_4_ NPs by coating the NP inner core with an exosomal membrane via the exosome biogenesis pathway (Reproduced with permission; copyright BioMed Central Ltd. (2020) [202]). **b** Schematic of the exosome spray method and fabrication process (Reproduced with permission; copyright American Chemical Society (2021) [214]). IV intravenous, MI myocardial infarction, MSC mesenchymal stem cell, NPs nanoparticles

The wound-healing effects of MSC-derived exosomes were investigated in vivo by camouflaging superparamagnetic iron oxide NPs (Fe_3_O_4_ NPs) with MSC-derived exosomal membranes. This was achieved by the direct incubation of Fe_3_O_4_ NPs with MSCs. MSC-secreted exosomes contain exogenous NPs via the exosome biogenesis pathway (Fig. 8a) [202]. Because of the restricted ability of MSC-derived exosomes to target wounded skin sites, magnetic guidance was employed to efficiently deliver exosomal-membrane-coated Fe_3_O_4_ NPs to injured skin. Owing to their magnetic properties, the Fe_3_O_4_ cores enhanced the targeting ability of MSC-derived exosomes to the wounded skin sites of mice after intravenous (IV) injection. Treatment with exosomal-membrane-coated Fe_3_O_4_ NPs using magnetic guidance enhanced collagen synthesis and re-epithelialization, accelerated wound closure, and reduced scar formation, which resulted in the up-regulation of skin healing-associated proteins, such as cyclin A2, cyclin D1, VEGFA, and C-X-C motif chemokine 12. In summary, the pro-angiogenic effects of the exosome-mimicking nanosystem were 2-fold higher than those of non-coated NPs, leading to a significant reduction in the area of the injured skin after 3 and 5 weeks [202].

Exosome-based therapy is a novel method for restoring bone defects without the use of cells. This therapeutic regime is based on cell–cell communication mediated by exosomes for the transfer of genetic materials and critical proteins. When compared to routine methods for bone defect restoration that require cell transplantation, cell-free exosome-based therapy is advantageous in reducing cell accumulation within the organ (e.g., the liver). Other benefits include an intrinsic homing effect, considerable chemical and physical stability, and low immunogenicity. Human adipose-derived stem cells (hASCs) undergo rapid osteogenic differentiation both in vitro and in vivo. The use of hASC-derived exosomes further accelerates angiogenesis and enables cation transfer and incorporation of cations into bone defects. In addition, hASC-derived exosomes have been shown to enhance the proliferation, migration, and osteogenic differentiation of MSCs both in vitro and in vivo. These properties render hASC-derived exosomes a suitable candidate and a promising alternative for future clinical trials [213].

As mentioned in the previous section, strategies involving cell transplantation have considerable drawbacks, such as their risks of tumorigenesis and immunogenicity. Therefore, an acellular approach has emerged based on the stem cell-derived secretome and its associated exosomes. Recently, scientists have investigated minimally invasive, sprayable cardiac patches based on MSC-derived exosomes [214]. These patches were used to create the product “exosome spray” (EXOS) by combining with a fibrin sealant to generate gelation properties (Fig. 8b). The fibrin scaffold was approved by the US Food and Drug Administration and was characterized using scanning electron microscopy. This invention can be used as an alternative to open surgery, which can result in severe physical trauma. EXOS increases the retention of MSC-derived exosomes in the heart even after MI, and increases the uptake of these exosomes by cardiomyocytes. This increased uptake leads to a reduction in cell apoptosis and an increase in the cell proliferation rate. In vivo experiments showed that EXOS reduced infarct size, improved cardiac function, preserved viable cardiac tissue cells, and increased ventricular wall thickness. Additional experiments have shown that EXOS is capable of improving angiomyogenesis after MI [214].

A recent study has shown that natural exosomes can mediate both the expression and transfer of genetic materials and vital proteins after their secretion from cells [215]. This is promising for different tissue engineering applications, as well as for promoting gene expression within targeted organs and tissues. The delivery of genetic materials and vital proteins to targeted organs and tissues may be enhanced by decorating the surface of exosomes with siRNA. The inclusion of siRNA on the exosomal surface protects the cargo, increases the targeted delivery ratio, and decreases off-target effects. This approach may be beneficial for myocardial regeneration. Scientists have found that genetically decorated exosomes derived from bone marrow stromal cells considerably improve tube formation from human umbilical vein endothelial cells. This strategy inhibits the proliferation of T cells in vitro and in vivo [216].

### Neurodegenerative diseases

The most serious challenge in the diagnosis and treatment of neurodegenerative disorders is the difficulty of drug delivery systems in crossing the blood–brain barrier and targeting neuronal cells. Exosomal-membrane-coated NPs have been used to image and treat neurodegenerative disorders. For instance, Au NPs have been functionalized with neuron-targeting exosomes derived from genetically engineered human embryonic kidney cells (HEK293T). Coating Au NPs with exosomal membranes enhances their penetration through the blood–brain barrier and improves their accumulation in neuronal cells [217]. Exosomal membranes have been conjugated with neuron-targeting ligands such as the rabies virus glycoprotein (RVG) peptide. This combination improves the brain-targeting ability of exosomes because of the specific binding of the RVG peptide to acetylcholine receptors expressed by neuronal cells. To harness the potential benefits of such a strategy, exosome-producing HEK293T cells were transfected to produce exosomes with RVG peptides on their surfaces. The modified exosomes were then isolated from the cell-culture supernatant and used to coat Au NPs. The ability of Lamp2b-RVG and glycosylation-stabilized peptide-decorated Au NPs to penetrate the blood–brain barrier and specifically target brain cells was demonstrated both in vitro and in vivo after IV injection in a mouse model by bioluminescent imaging of the mouse brain. When compared to Au NPs coated with non-RVG-targeted exosomes, Au NPs coated with RVG-targeted exosomes were more efficacious in crossing the blood–brain barrier and accumulated more abundantly in brain cells. In vitro, a penetration rate of 20% across the blood–brain barrier was achieved 24 h after incubation, which was considerably higher than that of non-RVG-targeted exosome-coated Au NPs. This study reveals a promising approach to overcoming the challenge of crossing the blood–brain barrier, and pioneers the development of effective diagnostic and treatment strategies for various brain diseases [217].

AD is the most prevalent form of dementia worldwide. Globally, the number of people affected by this neurodegenerative disease is expected to increase considerably over the next few decades. AD is characterized by gradual memory loss and cognitive decline. Impairment of daily tasks occurs when patients lose their autonomy entirely [218]. The accumulation of amyloid beta peptides and hyperphosphorylated tau proteins in memory-associated areas of the brains of patients with AD results in the formation of amyloid plaques and neurofibrillary tangles, respectively. These aggregates are considered the two major histopathological hallmarks of the later stages of AD [219]. Cadmium (Cd) toxicity is associated with an increase in amyloid beta and phosphorylated tau protein levels, both of which are associated with AD. Furthermore, Cd exposure may contribute to AD due to degenerative brain alterations [220]. In a recent study investigating the potential of NPs and exosomes to ameliorate neurological disorders, copper sulfide NPs and MSC-derived exosomes were co-delivered to rats in a Cd-induced neurological disorder model [221]. Improved anticholinesterase, antioxidant, and anti-inflammatory responses were observed after IV injection of MSC-derived exosomes and copper sulfide NPs. Histological evaluations revealed that treatment with MSC-derived exosomes and copper sulfide NPs decreased the toxic effects of Cd on brain tissue and reduced degenerative alterations originating from neuronal disorders [221]. Future investigations should evaluate the applications of exosomal-membrane-coated NPs in the delivery of biomaterials, drugs, and genes.

PD is also a common neurodegenerative disease. It is characterized by the progressive loss of dopaminergic neurons, resulting in a dopamine deficit. In dopaminergic neurons, there is an abnormal accumulation of α-synuclein (α-syn). This protein is encoded by the synuclein alpha (*SNCA*) gene, which is the main component of Lewy bodies. Lewy body dementia is the most typical pathological manifestation of PD, and causes problems in thinking, movement, behavior, and mood [222]. Recently, a method was developed for reducing the expression and cytotoxicity of α-syn aggregates in dopaminergic neurons and delaying the progression of PD. This approach is based on the use of exosomal-membrane-coated NPs [223]. In this study, a biomimetic core–shell nanosystem was developed by co-loading phenylboronic acid-poly[2-(dimethylamino)ethyl acrylate] NPs with curcumin and siRNA targeting *SNCA*. The assembly core was subsequently coated with RVG-modified exosomal membranes derived from immature DCs. The biomimetic core–shell nanosystem effectively crossed the blood–brain barrier and targeted dopaminergic neurons. The loaded drugs were released into dopaminergic neurons in a reactive oxygen species-responsive manner to synergistically down-regulated α-syn synthesis and reduce existing α-syn aggregates. siRNA targeting *SNCA* inhibited α-syn aggregation by reducing α-syn synthesis, whereas curcumin directly reduced existing α-syn aggregates. Due to the synergistic effects of both drugs, the biomimetic nanosystem was more effective than its non-coated counterparts in clearing α-syn aggregates in dopaminergic neurons and in reducing *SNCA* mRNA expression (a 64% reduction was achieved). With the demonstration of improved neuronal repair and motor behavior in vivo, the biomimetic core–shell nanosystem has the potential for being used to effectively treat PD [223].

## Clinical translation and regulation

Exosome-based systems should be included under the designations “investigative medicinal products” (Europe) and “investigative new drugs” (US) [103]. Regarding the characterization and quality control of exosome-based products, the ISEV offers useful guidelines and explanations regarding 1) nomenclature, 2) exosome collection and pre-treatment, 3) exosome separation and purification, 4) exosome characterization, and 5) recommendations on functional studies to be performed [22].

Increasing knowledge of exosome functionality and biological roles has provided pivotal opportunities for the application of exosome-mimicking nanosystems in tissue repair, wound-healing, and the management of neurodegenerative diseases, among other applications. Despite significant advances in the development of next-generation exosome-based therapies, significant challenges prevent the leverage of these therapies in clinical settings, including the need for extensive and robust characterization, issues concerning large-scale production and reproducibility of exosome-related biomaterials (including exosomal-membrane-based nanosystems), standardization of manufacturing protocols, and the necessity to better understand the biodistribution and targeting features of exosomes [224].

Despite the excitement of exosomal-membrane-coating nanotechnology as a novel field of research, these challenges pose a significant hurdle for human clinical applications. Studies reporting the therapeutic potential of exosomal-membrane-coated NPs in tissue repair, wound-healing, and neurodegenerative diseases have not yet been scaled up to human clinical trials, being restricted to in vitro and in vivo mouse models.

## Challenges and future perspectives

Exosomal-membrane-coated NPs are emergent and promising nature-inspired delivery systems for biomedical applications. Although significant progress has been made in the field of exosomal-membrane-coating nanotechnology, this is a relatively new technological approach, and research in this area is still in its infancy. Enormous challenges currently hinder the implementation of exosomal-membrane-coated NPs in clinical settings, including 1) complex intra-exosomal composition, 2) heterogeneity, 3) reproducibility, 4) the lack of standardized methods for exosome isolation and purification, 5) the difficulty of large-scale manufacturing, 6) the lack of agreement over the ideal coating method, and 7) the high risk that the coating techniques may compromise the biological functions of natural exosomes and their safety profiles [18, 21]. Another critical issue faced by scientists is the current lack of understanding of the biogenesis, composition, and biological function of natural exosomes. To design exosomal-membrane-coated NPs more efficiently and safely, future research should focus on clarifying the complex composition, biological functionalities, and intrinsic targeting abilities of natural exosomes [18].

One major challenge when using natural, cell-secreted exosomes is exosome isolation and purity [22]. Various methods have been proposed for exosome isolation, including differential ultracentrifugation, density gradient ultracentrifugation, size-exclusion chromatography, and affinity/immunoaffinity capture [225]. All of these approaches have their own advantages and drawbacks, and thus far, there has been no standardization of the best isolation technique. Exosomes can be isolated from cell-culture supernatants or biological fluids such as plasma and serum. Each source has specific features that must be considered when isolating exosomes [22]. If exosomes are isolated from cells, one aspect to consider is the risk of isolation. Apart from cell-secreted exosomes, contaminant vesicles derived from fetal bovine serum (FBS) are often added to cell cultures. Precautions must be taken when using exosome-free FBS or bovine serum albumin instead of FBS [225], as exosomes isolated from plasma or serum are notorious for being contaminated with non-EV proteins (albumin and globulins) and non-EV lipidic structures (chylomicrons and lipoproteins), which can form non-EV particles [22]. Plasma is recommended over serum owing to the platelet EVs that are released during coagulation [226, 227]. Co-isolation of non-EV contaminants represents a major challenge for proper exosome isolation and analysis. Detailed information is needed regarding the isolation samples and their handling, namely storage and analytical procedures [22].

Different techniques have been investigated for coating NPs with exosomal membranes, with sonication and physical extrusion through porous membranes being the two most frequently used techniques. Another challenge for the clinical implementation of exosomal-membrane-coated NPs is related to the potential of coating methods to damage the integrity of the exosomal membrane’s structure and reduce its protein integrity. This may compromise the biological functions of natural exosomes and induce immunogenicity [18]. Exosomes contain a diverse set of proteins, some of which are responsible for their biological functions, whereas others may induce immune responses. Hence, manipulation of the exosomal membrane may modify the surface composition and orientation of these proteins. Such undue modifications may trigger immune responses and induce immunogenicity [18, 19]. There is an urgent need to develop new, non-disruptive coating techniques that do not adversely affect the protein integrity of the exosomal membrane or the efficacy and safety of biomimetic nanoplatforms [18].

Another major challenge is the lack of standardization regarding the best method for coating NPs with exosomal membranes [21]. It is generally accepted that the ideal coating method depends on the NP and cell types. Accordingly, studies should be performed using different types of NPs, progenitor cells, and coating methods to evaluate which encapsulation method is most favorable for a particular scenario [21].

The reduced number of exosomes naturally secreted by most cells and the current lack of standardized protocols for exosome isolation and purification represent major challenges for the successful implementation of natural exosomes in clinical settings and exosome production at a clinical scale [21]. Similar to natural exosomes, the clinical-scale production of membrane-coated NPs remains a significant obstacle. To circumvent the large-scale manufacturing challenges of these biomimetic NPs, approaches normally used to produce exosomes on a large scale, such as the generation of cell-derived nanovesicles using extrusion through porous membranes, have recently been employed [18]. In a recent effort to prepare exosome-mimetic nanovesicles to encapsulate NPs, magnetic MSC-derived nanovesicles have been used to camouflage iron oxide NPs for the treatment of ischemic strokes. Iron oxide NPs were encapsulated in MSC-derived nanovesicles by extruding MSCs treated with iron oxide NPs through porous membranes. The final exosome-mimetic nanovesicles exhibited a 5.1-fold higher accumulation at sites of ischemic brain injury in a mouse model after IV injection and magnetic guidance when compared to those administered without an external magnetic field. The nanovesicles were capable of inducing angiogenesis, demonstrating anti-apoptotic and anti-inflammatory characteristics, substantially reducing infarct volume, and enhancing motor function [228].

Another concern is the safety profile of exosomal-membrane-coated NPs. Because these NPs contain biological materials, their quality control is of high importance. Therefore, stringent investigation of the immunogenicity profiles and potential side effects of exosomal-membrane-coated NPs should be determined prior to their translation into clinical settings [18, 19, 21]. In the future, to reduce potential undesirable immune responses and ensure the biosafety of these biomimetic nanoplatforms, the development of personalized therapy that utilizes the patient’s own exosomes to camouflage NPs should be investigated [21].

## Conclusions

The use of exosomal membranes to camouflage nanomaterials for biomedical applications is an attractive and promising technological approach because of their enhanced biocompatibility, non-immunogenicity, immune evasion abilities, prolonged blood circulation, intrinsic tissue-specific homing features, and cell-specific uptake [21]. Despite the enormous potential of exosomal-membrane-coated NPs for the targeted delivery of therapeutic and imaging molecules to sites of interest, this is a relatively new technological approach. Major challenges must be addressed before clinical translation can come to fruition. In recent years, research on this biomimetic approach is expected to continue to grow, which will enable the development of promising next-generation bioinspired nanosystems for a variety of biomedical applications with the potential to revolutionize the diagnosis and treatment of human diseases.

## Data Availability

Not applicable.

## Abbreviations

AD: Alzheimer’s disease
Cd: Cadmium
Cx43: Connexin 43
DC: Dendritic cell
DNA: Deoxyribonucleic acid
ESCRT: Endosomal sorting complex required for transport
EV: Extracellular vesicle
EXOS: “Exosome spray”
FBS: Fetal bovine serum
hASC: Human adipose-derived stem cell
Hsps: Heat shock proteins
ISEV: International Society for Extracellular Vesicles
IV: Intravenous
Lamp2b: Lysosome-associated membrane glycoprotein 2b
MI: Myocardial infarction
miRNA: MicroRNA
MISEV: Minimal Information for Studies of Extracellular Vesicles
mRNA: Messenger RNA
MSC: Mesenchymal stem cell
MVB: Multivesicular body
NP: Nanoparticle
PD: Parkinson’s disease
PLGA: Poly(lactic-*co*-glycolic acid)
RNA: Ribonucleic acid
RVG: Rabies virus glycoprotein
siRNA: Small interfering RNA
VEGF: Vascular endothelial growth factor
α-syn: α-Synuclein
